# Mild Cognitive Impairment: Beyond Memory Dysfunction

**DOI:** 10.1155/2012/262305

**Published:** 2012-07-17

**Authors:** Andrea Tales, Antony Bayer, Štefan Krajčík, Kurt A. Jellinger

**Affiliations:** ^1^School of Experimental Psychology, University of Bristol, 12a Priory Road, Clifton, Bristol BS8 1TN, UK; ^2^Cochrane Institute of Primary Care and Public Health, School of Medicine, Cardiff University, Cardiff CF64 1AT, UK; ^3^Geriatric Department, Slovak Medical University, Krajinska 91, 825 56 Bratislava, Slovakia; ^4^Institute of Clinical Neurobiology, Kenyongasse 18, 1070 Vienna, Austria

Traditionally, mild cognitive impairment (MCI) has tended to be primarily characterised and diagnosed in relation to the integrity of amnestic function and thus studied accordingly. However, a more recent multidisciplinary and collaborative research approach has investigated a much wider range of cognitive, functional, structural, and behavioural integrity and is revealing an extensive and often complex range of characteristics in addition to memory dysfunction that differentiate MCI from healthy ageing. These may be of especial relevance when considering MCI as a potential prodromal stage of dementia. 

Several papers in this special issue report novel and important findings with respect to the search for predictors of development of dementia in patients with MCI. 

The paper by E. L. Abner et al. describes a new approach in the assessment of risk factors for dementia, including age, gender, education, apolipoprotein E status, family history of dementing illness, and baseline hypertension. Their results highlight the importance of objective criteria in MCI diagnosis. 

I. Reinvang et al. report on how studies of genetic high-risk groups, using sensitive cognitive neuroscience paradigms, indicate the potential for differences in executive function to be a cognitive marker useful for tracking development of the pathophysiological changes of Alzheimer's disease (AD).

The paper by D. V. Moretti et al. reveals specific electroencephalographic (EEG) changes associated with atrophy of the hippocampus in people with MCI and AD, namely, that the increase of alpha3/alpha2 power ratio is correlated with atrophy of the hippocampus both in MCI and in AD patients, suggesting a possible diagnostic role of EEG markers.

In a comprehensive review, B. Ferencz et al. provide an overview of brain changes in early AD and MCI, together with evidence for recent advances in neuroimaging and genetic biomarkers and the importance of altered mitochondrial dynamics in the preclinical stages of AD.

D. P. Devanand et al. discuss the outcome of their detailed and novel study that uses clinical and magnetic resonance imaging (MRI) variables to compare predictor models for the transition to AD in patients with MCI. 

Research priority has until relatively recently been directed to the amnestic type of mild cognitive impairment (aMCI). However, the paper by A. Poggesi et al. provides an important overview of the concept of vascular MCI and how it may signal the prodromal stages of vascular dementia or the presence of small vessel disease. 

It is of course important to raise awareness of MCI within the public at large and also to determine the factors likely to encourage individuals to report a change in their memory to their general practitioner. C. Pires et al. highlight the importance of the type of memory complaint in determining whether an individual seeks medical attention and describe how forgetting the name of family members or friends is a particularly strong impetus for such contact.

Increasingly, studies are addressing the potential of MCI to adversely affect a wide range of factors that impinge upon an individual's quality of life. In the paper by G. Arsenault-Lapierre et al., the importance of stress in the lives of those with MCI and dementia is described, together with the finding that patients with MCI or dementia can display anosognosia, which can preclude patients from accurately appraising their own level of stress.

Together these papers contribute to our further understanding of the concept of MCI and of its potential progression to dementia. All highlight the need to think beyond merely memory dysfunction in MCI and to continue to expand our knowledge in this important field of scientific and clinical research.

*Andrea Tales* *Andrea Tales* *Antony Bayer* *Antony Bayer* *Štefan Krajčík* *Štefan Krajčík* *Kurt A. Jellinger* *Kurt A. Jellinger*

